# Supplementary material for: Geographic Differences in Phenotype and Treatment of Children with Sickle Cell Anemia from the Multinational DOVE Study
Source: J Clin Med. 2019 Nov 17;8(11):2009. doi: 10.3390/jcm8112009 (PMC6912763; doi:10.3390/jcm8112009)
Supplement: Supplementary file 1 [file jcm-08-02009-s001.pdf]

SUPPLEMENTARY MATERIAL

SUPPLEMENTARY TABLE S1 Baseline blood pressure by geographic region and age group.

	Geographic Regions[a]										
	SSA		Americas		North Africa/ Middle East		Europe		Total		p-Value
	N	mmHg	N	mmHg	N	mmHg	N	mmHg	N	mmHg	
**Systolic Blood Pressure [Mean (SD)]**											
2 to <6 years	32	93.3 (8.8)	9	101.8 (8.0)	17	97.5 (10.2)	9	98.6 (7.4)	67	96.2 (9.2)	0.317
6 to <12 years	72	98.8 (8.5)	22	107.0 (10.0)	29	100.5 (12.0)	9	102.1 (9.9)	132	100.8 (10.1)	**0.018**
12 to <18 years	44	103.3 (10.2)	26	111.0 (11.4)	64	109.6 (10.1)	8	117.4 (9.9)	142	108.4 (10.9)	0.079
**Diastolic Blood Pressure [Mean (SD)]**											
2 to <6 years	32	54.8 (8.3)	9	62.9 (6.2)	17	60.9 (10.0)	9	56.6 (7.6)	67	57.7 (8.9)	0.106
6 to <12 years	72	58.8 (6.2)	22	61.5 (8.7)	29	58.7 (8.3)	9	60.4 (6.8)	132	59.3 (7.2)	0.112
12 to <18 years	44	60.0 (5.9)	26	60.2 (6.4)	64	65.4 (9.1)	8	64.8 (10.1)	142	62.8 (8.2)	**0.004**

Abbreviations: N=number of randomized subjects; SD=standard deviation; SSA=sub-Saharan Africa.

[a] The SSA subgroup includes Ghana and Kenya; the Americas subgroup includes Brazil, the United States, and Canada; the North Africa/Middle East subgroup includes Saudi Arabia, Oman, Egypt, Lebanon, and Turkey; the Europe subgroup includes Belgium, Italy, and the United Kingdom (Heeney et al.).[9]

SUPPLEMENTARY TABLE S2 Mean baseline laboratory values for subjects from East Africa (Kenya) and West Africa (Ghana) not on HU.

Parameter Assessed [Mean (SD)]	Ghana (n=56)	Kenya (n=75)	p-Value
Total Bilirubin (µM/L)	35.3 (19.8)	42.2 (31.5)	**0.007**
Hemoglobin gL)	49 (0.6)	45 (0.6)	**<0.001**
Lactic Dehydrogenase (units/L)	576.1 (151.4)	564.7 (166.9)	0.896
Mean Cell Volume (fL)	84.8 (9.3)	84.6 (10.8)	0.805
Platelet Volume (fL)	9.1 (0.8)	8.8 (0.7)	0.207
Platelet Count (x10^9/L)	373.6 (110.9)	490.4 (142.2)	**<0.001**
Reticulocyte Count (x10^9/L)	278.4 (119.2)	349.8 (107.6)	**0.002**
Leukocyte Count (x10^9/L)	13.6 (3.5)	16.1 (4.8)	**0.029**

Abbreviations: HU = hydroxyurea; n=number of subjects with at least one parameter measured at baseline; SD=standard deviation.
